# Role of Nuclear-Receptor-Related 1 in the Synergistic Neuroprotective Effect of Umbilical Cord Blood and Erythropoietin Combination Therapy in Hypoxic Ischemic Encephalopathy

**DOI:** 10.3390/ijms23052900

**Published:** 2022-03-07

**Authors:** Joo-Wan Choi, Su Jung Kang, Jee In Choi, KyuBum Kwack, MinYoung Kim

**Affiliations:** 1 Department of Rehabilitation Medicine, CHA Bundang Medical Center, CHA University School of Medicine, Seongnam 13496, Gyeonggi-do, Korea; bubukkum@naver.com (J.-W.C.); cji-012@daum.net (J.I.C.); 2 Rehabilitation and Regeneration Research Center, CHA University, Seongnam 13496, Gyeonggi-do, Korea; 3 Department of Biomedical Science, College of Life Science, CHA University, Seongnam 13488, Gyeonggi-do, Korea; sujung_k@chauniv.ac.kr

**Keywords:** cerebral palsy, human cord blood cell therapy, erythropoietin, hypoxic-ischemic brain injury, comprehensive analysis, microarray, Wnt/β-catenin pathway, nuclear receptor related 1

## Abstract

Neonatal hypoxic–ischemic encephalopathy (HIE) results in neurological impairments; cell-based therapy has been suggested as a therapeutic avenue. Previous research has demonstrated the synergistically potentiated therapeutic efficacy of human umbilical cord blood (UCB) by combining recombinant human erythropoietin (EPO) treatment for recovery from HIE. However, its molecular mechanism is not entirely understood. In the present study, we analyzed the mechanisms underlying the effect of combination treatment with EPO and UCB by transcriptomic analysis, followed by gene enrichment analysis. Mouse HIE model of the neonate was prepared and randomly divided into five groups: sham, HIE, and UCB, EPO, and UCB+EPO treatments after HIE. A total of 376 genes were differentially expressed when |log2FC| ≥ 1-fold change expression values were considered to be differentially expressed between UCB+EPO and HIE. Further assessment through qRT-PCR and gene enrichment analysis confirmed the expression and correlation of its potential target, *Nurr1*, as an essential gene involved in the synergistic effect of the UCB+EPO combination. The results indicated the remarkable activation of Wnt/β-catenin signaling by reducing the infarct size by UCB+EPO treatment, accompanied by *Nurr1* activity. In conclusion, these findings suggest that the regulation of Nurr1 through the Wnt/β-catenin pathway exerts a synergistic neuroprotective effect in UCB and EPO combination treatment.

## 1. Introduction

Neonatal hypoxic–ischemic encephalopathy (HIE), which is typically caused by hypoxia during the perinatal period, may lead to death, intellectual disability, convulsion, seizure, paralysis, and long-term neuro-functional disabilities in children [1,2]. Approximately 20–50% of infants affected by a hypoxic–ischemic insult die during their postnatal period [3], and 25% go on to develop a significant neurodevelopmental disability, most commonly cerebral palsy (CP), epilepsy, motor and mental deficits, and cognitive disabilities [4]. Neonatal HIE is characterized by complex neuronal conditions, such as oxygen deprivation, inflammatory response, glutamate excitotoxicity, the production of oxygen free radicals, and predominantly necrotic cell death [5]. Although disability from HIE is detrimental, currently, there is no effective treatment, and cell therapy reportedly has therapeutic potential for HIE [6].

Various cell therapies for recovery from neurological impairments in HIE have been shown to be effective in preclinical studies [7,8,9]. Moreover, clinical trials of intravenous infusions of human umbilical cord blood (UCB) cells for cerebral palsy caused by HIE were demonstrated to be advantageous, with fairly stable safety [10]. However, due to insufficient evidence of efficacy that might rationalize its use, cell therapy of neuroregenerative purposes have not been applied in standard clinical practice [11,12]. To overcome this limitation, efforts and the co-administration of growth factor in cell therapy showed the possibility of potentiating efficacy [13]. Previous research has revealed the combined use of mesenchymal stem cells and recombinant human erythropoietin (EPO) for treating cerebral ischemia increased cell proliferation and neurogenesis [14,15]. Our clinical research team reported significantly improved outcomes only by the co-administration of UCB and EPO in children with cerebral palsy, whereas UCB administration resulted in inconsistent recovery [16]. When these treatments were applied to transient focal cerebral ischemia model in adult rats, UCB or EPO therapy alone demonstrated some neuroprotection; however, therapeutic effects were remarkably elevated by their co-administration. As a mechanism of this synergistic effect, neurogenesis promotion and angiogenesis were discovered [17].

In examining the mechanism of recovery from neuronal injury, previous experiments investigated target pathways that might be relevant to the therapy rather than screening all the molecular changes [18,19,20]. Microarray technology is powerful tool to elucidate the mechanisms within complex biological processes [21], providing essential theoretical and practical values in gene expression, genome research, and the intensity of hybridization signals of gene expression profiles [22]. Although this technique has been widely adopted to discover the differential expression of genes, to the best of our knowledge, no comparative studies of the therapeutic potential of UCB and EPO regarding whole-genome expression patterns in the host HIE brain have been performed. In this study, to develop a better understanding of synergistically activated neuroprotective mechanisms, microarray techniques were used to determine gene expression changes induced by UCB and EPO in HIE mouse model. Additionally, we performed confirmation experiments for the emerged genes through the microarray analysis.

## 2. Results

### 2.1. Comprehensive Analysis of Gene Expression Profiles in Each Treatment Sample after HIE

**Figure 1:**
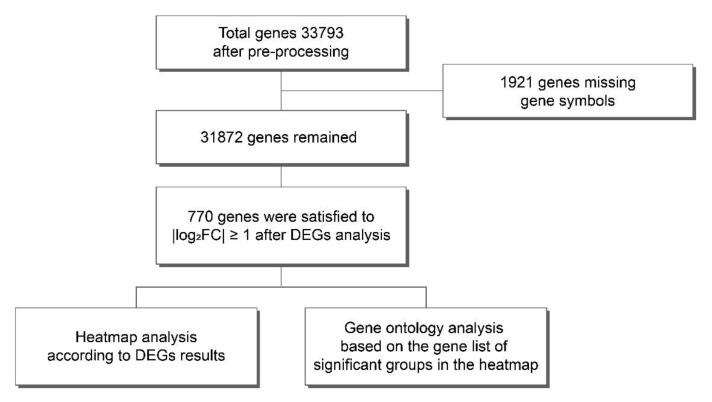
Design and workflow of the comprehensive analysis. Schematic overview of microarray gene identification pipeline summarizes all steps of the procedure.

The whole transcriptomes of each treatment sample of the HIE brain were analyzed by microarray. Total RNA was isolated from the brain. The changes in gene expression, according to each treatment from the gene expressions in the HIE sample, were assessed. Brain samples treated with EPO, UCB, and UCB+EPO one week after HIE injury, HIE, and sham injury control at the same age were used for analyses of differentially expressed genes (DEGs). One mouse from each group was randomly selected regardless of sex and used for RNA extraction for microarray testing. Male mice were allocated for sample of sham, UCB, and UCB+EPO treated after HIE injury, and female mice in the other groups. A total of 33,793 genes were detected after initial mapping followed by pretreatment, and 1921 genes were excluded due to probes that were not sufficiently annotated for further study. Then, genes with fold change expression values of |log2FC| ≥ 1 were considered as differentially expressed (Figure 1) [23]. As a result, 770 probes were identified as DEGs and subjected to further analysis (Supplementary Table S1).

In the next step, the DEG analysis was conducted to show the 30 most upregulated genes and 30 most downregulated genes (Supplementary Table S2). Five brain samples were analyzed for DEG and cut into the top 30 genes; then, the treated samples were compared with the HIE sample. Supplementary Table S2A–D present the upregulated genes, and Table S2E–H represent the downregulated genes; sham vs. HIE (Table S2A,E), EPO vs. HIE (Table S2B,F), UCB vs. HIE (Table S2C,G), and UCB+EPO vs. HIE (Table S2D,H).

### 2.2. Identified Pathways of Genes Upregulated in UCB+EPO Compared with HIE

**Figure 2:**
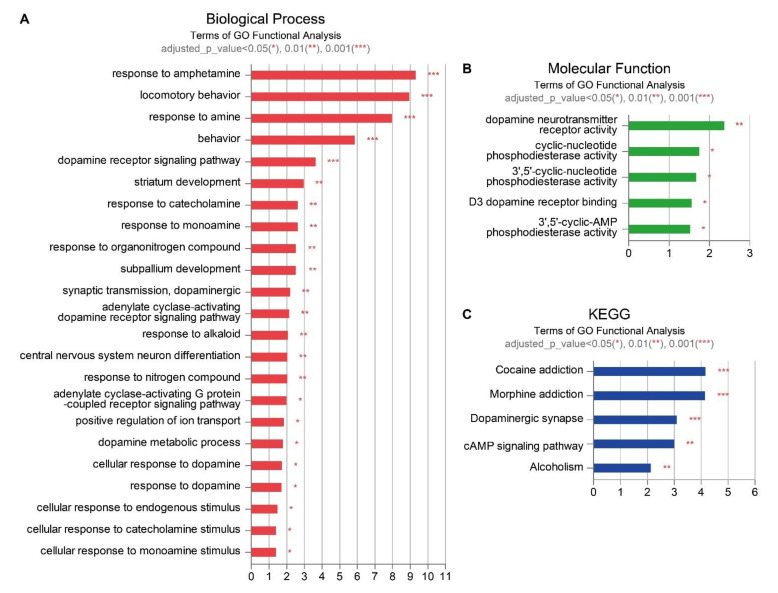
Various gene ontology of genes filtered by |log2FC| ≥ 1 in UCB+EPO vs. HIE among all genes by rich pathways in the g:profiler web. (**A**) Biological process terms.; (**B**) Molecular function terms; (**C**) KEGG pathway. The *X*-axis represents −log10 (adjusted *p*-value), and *Y*-axis represents the enriched terms. * *p* < 0.05, ** *p* < 0.01, and *** *p* < 0.001 significantly different from the HIE sample.

A list of genes with higher gene expression in the UCB+EPO-treated brain than in the HIE sample was identified among all the genes by enrichment pathways in the g:Profiler web tool (Figure 2) [24]. After applying Bonferroni correction for the *p*-value, only pathways with values less than 0.05 were extracted. Among the biological process (BP) terms, 23 terms, including responses to amphetamine, locomotory behavior, response to amines, behavior, and the dopamine receptor signaling pathway were derived (Figure 2A). Among the molecular function (MF) terms, five terms were found to be significant: dopamine neurotransmitter receptor activity, cyclic-nucleotide phosphodiesterase activity, 3’,5’-cyclic-nucletide phosphodiesterase activity, D3 dopamine receptor binding, and 3’,5’-cyclic-AMP phosphodiesterase activity (Figure 2B). In the KEGG pathway, five pathways were identified: cocaine addiction, morphine addiction, dopaminergic synapse, the cAMP signaling pathway, and alcoholism (Figure 2C).

### 2.3. Temporal Gene Expression Profiling the Effect of EPO or UCB or UCB+EPO Combination Treatment on the HIE Model

**Figure 3:**
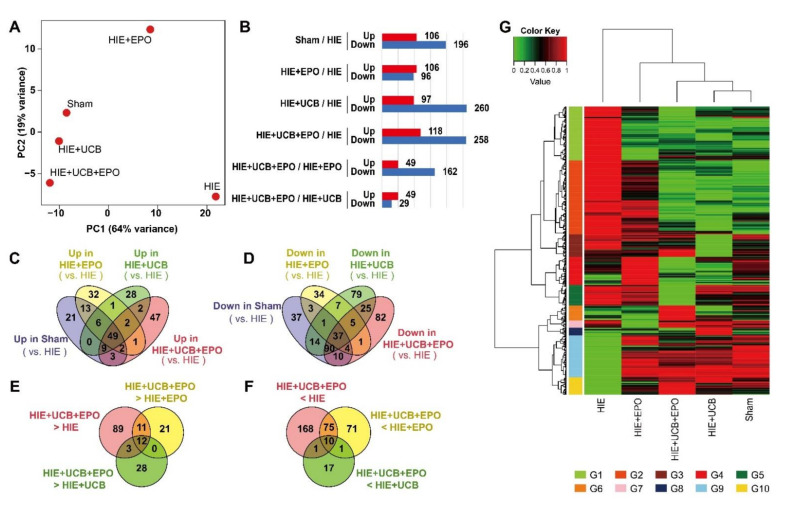
Comprehensive analysis of differential expressed genes (DEG) in each treatment brain sample after HIE. (**A**) The similarity of gene expression levels in the five samples was confirmed in principal component analysis plots. It has the explanatory power of PC1-64% (*X*-axis) and PC2-19% (*Y*-axis). (**B**) The DEG number of sham vs. HIE, EPO vs. HIE, UCB vs. HIE, UCB+EPO vs. HIE, UCB+EPO vs. EPO, and UCB+EPO vs. UCB. Histograms show the number of DEG that were up- or down-regulated in each sample. Venn diagram of DEGs showing overlap of (**C**) up-regulated and (**D**) down-regulated genes between sham vs. HIE, EPO vs. HIE, UCB vs. HIE, UCB+EPO vs. HIE, UCB+EPO vs. EPO, and UCB+EPO vs. UCB. Each colored ellipse on the Venn diagram represents the number of genes with high expression only for each sample and the number of genes with high expression common to each sample in each cross area. The Venn diagram of DEGs shows the overlap of (**E**) up-regulated and (**F**) down-regulated genes between UCB+EPO vs. HIE, EPO, and UCB treated brain samples. DEGs were identified as |log2FC| ≥ 1. (**G**) Unsupervised hierarchical clustering analysis of each brain sample in DEGs results. The *X*-axis lists the samples, and the *Y*-axis lists the genes in the DEGs results. Gene expression levels are converted to a number between 0 and 1 on a standard scale and expressed as a color gradient.

We investigated the gene expression profile of the brain sample of each treatment after HIE, HIE by itself, and sham control. A principal component analysis (PCA) plot of principal component 1 (PC1) and principal component 2 (PC2), which accounted for 83% of the total variation in the dataset (64% for PC1 and 19% for PC2), clustered from the five different samples. The PCA results exhibit closer clustering for more similar expression characteristics of the genes between the brain samples (Figure 3A) [25]. The numbers of DEGs in each sample are presented in the bar graph (Figure 3B). Genes with a |log2FC| ≥ 1-fold change expression value were considered to be differentially expressed. The Venn diagram represents the number of genes which were common in each treated sample, and the number of genes significant only in each sample (Figure 3C–F) [26]. There were 21, 32, 28, and 47 genes with higher expression levels compared with the HIE-only genes in the sham, EPO, UCB, and UCB+EPO groups, respectively. There were 37, 34, 79, and 82 genes with lower expression levels compared with the HIE-only genes in the sham, EPO, UCB, and UCB+EPO groups, respectively. There were 89, 21, and 28 genes with higher expression levels in UCB+EPO compared with genes in the HIE, EPO, and UCB, respectively. There were 168, 71, and 17 genes with lower expression levels in the UCB+EPO group Compared with genes in the HIE, EPO, and UCB groups, respectively. Expression patterns were visualized through the unsupervised hierarchical clustering analysis of each sample and gene. The *X*-axis shows the samples, and the *Y*-axis shows the genes in the DEG results. In the UCB+EPO sample, it was confirmed that the expression of the G6 clustering group was upregulated (Figure 3G) [27].

### 2.4. Gene Enrichment Analyses of the Effect of EPO or UCB or UCB+EPO Combination Treatments on the HIE Model

Gene enrichment analysis using a gene ontology (GO) method was performed to elucidate the mechanisms following UCB and EPO combination treatment. Enriched terms were determined using the ClueGO module of Cytoscape [28]. A hypergeometric test with Bonferroni step-down corrections was applied to identify the significance of enrichment, and a list of results matching *p*-values of less than 0.05 was obtained [29]. The *p*-values were converted to −log10 and quantified. We experimentally validated the changes in the mRNA expression of candidate genes *Nr4a2, Slc30a3, Sema7a*, and *Baiap2l1* in the upregulated G6 group through unsupervised hierarchical analysis (Table 1).

### 2.5. Neuroprotective Effect of UCB+EPO Treatment via the Activation of Nurr1 Which Is Related to the Wnt/β-Catenin Pathway

**Figure 4:**
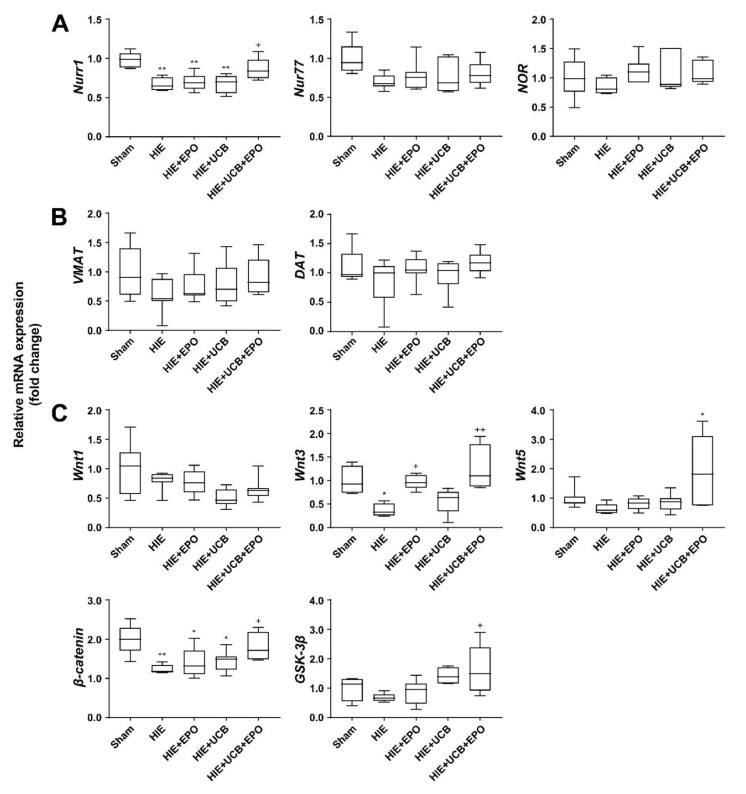
The combination of UCB and EPO treatment mainly activates the Nurr1-related Wnt/β-catenin signaling pathway—histograms of mRNA expression results for the representative Nurr1-related genes. The histogram shows the results of qRT-PCR for each group (sham, HIE, UCB, EPO, and UCB+EPO). The results of cycle threshold were calculated by the ΔΔ method to obtain the fold changes. Data are shown as the mean ± standard error of the mean. (**A**) The mRNA expression of *Nurr1*, *Nur77*, and *NOR* were detected by qRT-PCR in each treatment brain sample. Data are shown as the mean ± standard error of the mean. *n* = 4 to 6 per group, ** *p* < 0.01 vs. the sham group, and + *p* < 0.05 vs. the HIE group. (**B**) RT-PCR analysis of Nurr1-related dopaminergic pathway molecule (*VMAT* and *DAT*) expression for each group (sham, HIE, UCB, EPO, and UCB+EPO). (**C**) RT-PCR analysis of Nurr1-related pathway molecule (*β-catenin, GSK3β, p-GSK3β, Wnt1, Wnt3*, and *Wnt5*) expression for each group (sham, HIE, UCB, EPO, and UCB+EPO). GAPDH was used as an internal control. Data are shown as the mean ± standard error of the mean. * *p* < 0.05, ** *p* < 0.01 vs. the sham group, + *p* < 0.05, and ++ *p* < 0.01 vs. the HIE group (one-way ANOVA).

Among these genes, *Nr4a2 (Nurr1)* was selected as a representative gene that was changed according to the combination treatment of UCB and EPO. The mRNA expression of *Nurr1* was significantly higher (1.42-fold greater) in the UCB+EPO brain compared with the HIE sample (*p* < 0.05), whereas each treatment with UCB or EPO alone were similar to HIE (Figure 4A). To investigate whether the protective effect of UCB and EPO combination therapy was induced by the activation of Nurr1, we performed confirmation experiments. The expression of the NR4A nuclear receptor family, comprising *NR4A1 (Nur77)* and *NR4A3 (NOR)*, was quantified by qRT-PCR in brain tissue of the HIE model at P21 [30]. *Nur77* and *NOR* mRNA expressions did not reveal significant findings, and only *Nurr1* represented a significant result. Nurr1 is known to regulate the transcription of crucial dopamine-related genes, including tyrosine hydroxylase (TH), dopamine transporter (DAT), and vesicular monoamine transporter (VMAT), which significantly influence striatal dopamine levels [31,32]. To determine the molecular mechanisms of synergistic effects via the regulation of Nurr1 in UCB and EPO combination therapy, we examined crucial dopamine-related genes, such as VMAT and DAT. However, the levels of *VMAT* and *DAT* did not show differences among the groups (Figure 4B). The canonical Wnt signaling pathway and its crucial mediator β-catenin were reported to be essential regulators of functional maintenance of dopaminergic neurons in the brain [33]. Nurr1 is responsible for neuron development and maintenance. To confirm that the protective effect of UCB and EPO combination therapy is associated with the activation of Nurr1 through the Wnt/β-catenin pathway, we evaluated the mRNA expression of *Wnt1, Wnt3, Wnt5, β-catenin*, and *GSK-3β*. The mRNA expressions of *Wnt1, Wnt3, β-catenin*, and *GSK-3β* were significantly increased in the UCB+EPO group (*p* < 0.05) (Figure 4C).

### 2.6. Countervailed Neuroprotective Effect of UCB+EPO Combination Treatment by Inhibition of the Wnt/β-Catenin Pathway

**Figure 5:**
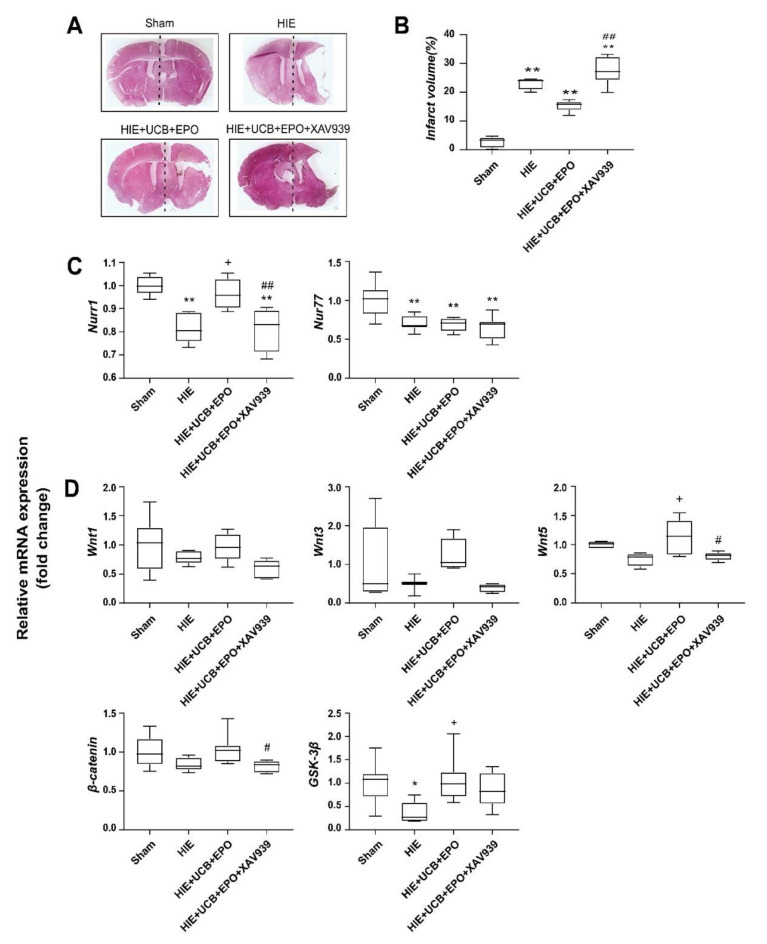
Inhibition of the wnt/β-catenin pathway by XAV939 suppresses the protective effect of the combination of UCB and EPO treatment after HIE. (**A**) Whole-brain sections were stained with Hematoxylin and eosin. (**B**) The graph depicts the total volume of the infarct size in the affected hemisphere, which was estimated compared to that in the contralateral side by the ImageJ program. Data are shown as the mean ± standard error of the mean. *N* = 3 per group * and + *p* < 0.05 (one-way ANOVA). (**C**) The mRNA expression of *Nurr1* and *Nur77* was detected by qRT-PCR in each treatment brain sample. (**D**) qRT-PCR analysis of Nurr1-related pathway molecule (*Wnt1, Wnt3, Wnt5, β-catenin*, and *GSK3β*) expression for each group (sham, HIE, UCB, EPO, and UCB+EPO). GAPDH was used as an internal control. *n* = 6 per group. Data are shown as the mean ± standard error of the mean. * *p* < 0.05, ** *p* < 0.01 vs. the sham group, + *p* < 0.05 vs. the HIE group, # *p* < 0.05, and ## *p* < 0.01 vs. the HIE group (one-way ANOVA).

To further explore a link between the Wnt/β-catenin pathway and the synergistic effect of UCB+EPO, we identified the protective effect of UCB+EPO when inhibiting the Wnt/β-catenin pathway. XAV-939, a Wnt/β-catenin inhibitor [34], was injected before the administration of UCB and EPO. As a result, brain damage was robustly reversed by XAV-939 pre-treatment in the brain of HIE mice that received UCB and EPO combination therapy (*p* < 0.05) (Figure 5A,B). Additionally, the level of Nurr1 was significantly increased by UCB and EPO combination therapy, and was also robustly reversed by XAV-939 pre-treatment (*p* < 0.05) (Figure 5C). Expressions of β-catenin and GSK-3β were significantly decreased by XAV-939 pre-treatment (*p* < 0.05) (Figure 5D). The finding indicates that the effect of UCB and EPO combination therapy relates to the Nurr1-related Wnt/β-catenin pathway and protects brain damage from HIE.

### 2.7. Analysis for Evaluating Effect Caused by Gender Difference

Six of the 770 DEGs overlapped as sexually dimorphic genes in neonatal mouse cortex and hippocampus [35]. Expressions of *Ctsz, Ddx3y, Eif2s3y*, and *Kdm5d* were male-biased, while those of *Fxyd5* and *Xist* were female-biased. *UTY* and *UTX (Kdm6a)*, the ubiquitous transcribed tetratricopeptide repeat genes are encoded on the Y and the X chromosome, respectively [36]. In these results, the *UTY* showed higher (3–4 fold) expression in male mice than in female mice, while *Kdm6a* did not show differential expressions among 770 DEGs (Supplementary Table S2). Ignoring the treatment method and comparing the expression level between the same sexes, there is no gene with a more than 2-fold difference in expression among the seven DEGs.

## 3. Discussion

In the present study, microarray analysis was used to detect the genes pivotal in transcriptomic changes associated with the synergistic effect of UCB and EPO treatment in an experimental neonatal HIE model. Our previous research revealed the synergistic effect of UCB and EPO combination treatment in HIE by ameliorating pro-inflammatory states and anti-apoptosis, with increases in the phosphorylation of Akt which regulates Bax and Bcl-2 [37]. Here, the authors specifically focused on the temporal expression profiling of a wide range of cellular activities, including the survival, proliferation, metabolism, and motility of cells using microarray data. Additionally, the gene that was derived as a result was confirmed with qRT-PCR. We examined a total of 31,873 genes; 770 DEGs were considered for further studies (|log2FC| ≥ 1) (Figure 1). GO analysis was used to assess the DEGs in terms of their enrichment BP terms and cellular component categories in the synergistic effect of UCB and EPO. The transcriptome analyses showed a similar tendency in the gene expressions of sham control and UCB+EPO-treated brains, which had higher association than that of cases treated with only UCB (Figure 2).

Interestingly, most of the significant BP terms in this study were associated with synaptic-related responses. Various MF terms detected in this study were related to dopamine activity. Recently, it was reported that the mesenchymal stem cell could be induced to differentiate neuronal/dopaminergic differentiation [38]. Additionally, other reports demonstrated improvements in dopaminergic function from administering embryonic stem-cell-derived neuron-like cells that accompanied neurobehavioral recovery in a cerebral ischemic model [39]. Therefore, we could draw one conclusion, that the efficacy of UCB and EPO was potentiated by combining these via improvements in dopaminergic function (Figure 3). GO analysis revealed possible involvement of the ‘dopamine-related pathway’, ‘zinc protein pathway’, ‘immune response’, and ‘cellular structure’ in BPs, in terms of the efficacy of UCB+EPO combination treatment, by discovering significant genes: *Nr4a2* (*Nurr1*), *Slc30a3*, *Sema7a*, and *Baiap2l1* (Table 1). Among them, Nurr1 reportedly provides significant neuroprotection against inflammation [40,41]. In a neonatal mouse model of HIE, a mutation of Nurr1 was revealed to exacerbate brain injury in infants with HIE [42]. After the verifying experiments with qRT-PCR in the present study, we only found *Nurr1* to be significant among the NR4A nuclear receptor family, with levels only elevated by UCB+EPO treatment and not by EPO or UCB treatment alone (Figure 4A). According to previous research, the efficacy of human cord blood-derived multipotent stem cell treatment appeared to be induced by Nurr1 activation [43]. In short, the results suggest a synergistically potentiated neuroprotective effect by hUCB and rhEPO combination treatment driven by *Nurr1*. Additionally, although we did not proceed with confirmation experiments for *Slc30a3*, it was reported to have neuroprotective effects on oxidative stress, which might have contributed to the therapeutic efficacy [44].

Nurr1 is related to the development, function, and maintenance of dopaminergic neurons [30]. A recent study showed that increased Nurr1 expression alleviated the infarct volume and improved the neurological outcomes in an acute stroke model [45]. Furthermore, Nurr1 is known to regulate the transcription of dopamine-related pivotal genes including tyrosine hydroxylase, DAT, and VMAT, which significantly influence striatal dopamine levels [46]. DAT or VMAT knock-out mice exhibited an aberrant neurotransmission of monoamines, resulting in starvation and early death [47]. However, the present study failed to show the significance of *DAT* and *VMAT*, although the *VMAT* results showed a similar tendency without significance. Instead, the authors found a crucial signaling pathway related to *Nurr1* activation, Wnt/β-catenin, which is vital in maintaining synaptic structure and neuronal functions (Figure 4B,C) [48]. Inhibition of the Wnt-signaling pathway was associated with several brain pathologies, such as schizophrenia and Alzheimer’s disease [33]. β-catenin is a component of tight endothelial junctions, and its loss may reduce junction stability and increase vascular permeability. This study found that HIE caused downregulations of *Wnt3* and *β-catenin* mRNA expression. *Wnt1, Wnt5*, and *GSK-3β* showed a tendency to degrade the HIE brain tissue, although without significance. UCB and EPO co-administration markedly increased mRNA expressions of *Wnt3, Wnt5, β-catenin,* and *GSK-3β*. To clarify the involvement of Nurr1 in Wnt/β-catenin-mediated signaling, we conducted an inhibition test using XAV939, an inhibitor of the Wnt/β-catenin pathway [34], for the UCB+EPO-administered HIE mice. The results revealed the nullified neuroprotective effects of UCB+EPO treatment by XAV939 on infarct volume. mRNA expressions of *Nurr1, Wnt5*, and *β-catenin,* and of *Wnt1, Wnt3*, and *GSK-3β*, also showed similar traits (Figure 5).

Taken together, we can conclude that microarray analysis is a powerful tool for detecting the mRNA expression pattern of many genes. The role of *Nurr1* in the synergistic neuroprotective effects of UCB and EPO combination treatment in HIE was revealed. Additionally, Wnt/β-catenin-mediated signaling was found to be associated with this mechanism. However, there are some limitations to the present study. We had a relatively limited sample size, which may have elevated the risk of false-positive results. This study did not analyze for the potential effect of sex. Although our previous investigation revealed neuronal recovery by UCB+EPO in both males and females [37], sexual dimorphism was reported regarding neuronal cell death by different mechanisms [49]. Recently, it was reported that long noncoding RNA XIST participates in hypoxia-induced angiogenesis in human brain microvascular endothelial cells through regulating miR-485/SOX7 axis [50]. Although no genes were found with a more than 2-fold difference in expression among the known seven genes linked with sexes, identical experiments must be performed separately on female and male mice to exclude gene expression differences caused by sex. Additionally, this study lacked Western blot experiment verification for the genes. Although we found a relationship between Nurr1 and Wnt/β-catenin signaling, the downstream effect that directly affects neuronal integrity was not investigated. Beside this UCB+EPO combination treatment, there can be various strategies, such as cooling and EPO and UCB application after cooling for neonatal HIE [51,52]. Actually, many studies have suggested potential therapeutic effect of combining hypothermia and stem cell therapy [53,54].

In conclusion, our findings provide comprehensive analysis results of DEGs and the associated signaling pathways induced by treatments in HIE. According to the gene screening and confirmation experiments, regulation of Nurr1 through the Wnt/β-catenin pathway may have exerted synergistic effects of UCB and EPO combination therapy.

## 4. Materials and Methods

### 4.1. Experimental Animals

Pregnant ICR mice were purchased from Koatech (Pyeongtaek, Korea). All the animal experiments were performed according to international guidelines and approved by the Institutional Animal Care and Use Committee (IACUC 200216) of CHA University. The mice were housed under controlled conditions, with free access to food and water, at 23 ± 2 °C, with 12 h light/dark periods.

### 4.2. Hypoxic Ischemic Encephalopathy (HIE) Model

At P7, pups whose sex was randomly selected were initially anesthetized with 3–5% isoflurane and maintained with 1–2% isoflurane for surgery. Briefly, a midline cervical incision was made in the anterior neck. The right common carotid artery was dissected and permanently ligated with a 5-0 blue nylon. The operated pups were exposed to hypoxic conditions in 8% O_2_ and 92% N_2_. Following exposure, the pups were returned to the cages with the dams. Sham-operated pups received anesthesia, and the artery was exposed without ligation or hypoxia.

### 4.3. Human Umbilical Cord Blood (UCB) Isolation

Human UCB was provided by the CHA Cord Blood Cell Bank. The cryopreservation of donated UCB was performed following the protocol of the facility. The mononuclear cells and plasma were obtained from UCB, isolated using density differences for Ficoll–Hypaque (GE healthcare, Chicago, IL, USA). The mononuclear cell concentrates were washed with dextran 40 and albumin before freezing. Then, the isolated mononuclear cell and plasma were cryopreserved with dimethylsulfoxide at −198 °C in a controlled-rate freezer. Prior to infusion, DMSO was thawed in a 37 °C water bath, from which DMSO used as cryoprotectant was washed using phosphate-buffered saline (PBS, Welgene, Daegu, Korea).

### 4.4. Experimental Design

**Figure 6:**
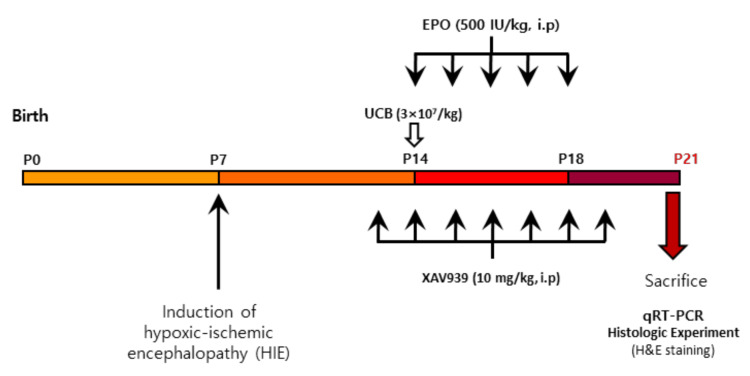
Experimental designs. Postnatal day 7 (P7) mice were induced with hypoxic-ischemic encephalopathy. On P14, the UCB (3 × 10^7^/kg) was injected, administered via intraperitoneal injection, and EPO (500 IU/kg) was given intraperitoneally from P14 for consecutive days. XAV939 (10 mg/kg) was given intraperitoneally from P14 for 7 consecutive days, 1 h before UCB and EPO treatment. One week after injection, the mice were sacrificed for qRT-PCR, Western blot, and microarray.

The number of total nucleated cells of UCB and the dosage of EPO used was based on our previous clinical trials [55,56], which was performed in patients with traumatic brain injury and cerebral palsy, and is considered safe for humans. At 7 days post-injury, the operated pups were randomly assigned into five groups (each group, *n* = 8 to 10). UCB (3 × 10^7^/kg, intraperitoneal injection once at 7 days post-injury), EPO (LGchem, Daejeon, Korea) (500 IU/kg, intraperitoneal injection for 5 consecutive days from 7 days HIE), UCB+EPO (at the same dose and schedule as the other groups), and UCB+EPO+XAV939 (Sigma, St. Louis, MO, USA) (10 mg/kg, 5 consecutive days from 7 days HIE) were intraperitoneally administered, and PBS (5 consecutive days from 7 days HIE) was injected as a control (HIE). The time for mRNA quantification was determined as 7 days after administration of UCB or initiation of EPO injection. Our previous experiment, where neurobehavioral outcome was assessed on P42 with significant findings, did not show differences in mRNA expression at that time [37]. The behavioral outcomes might have resulted long-after the preceded changes in the brain during several days after HIE or treatment for it [57]. Therefore, we regarded assessment of responses during the earlier stages to represent the key mechanism. The scheme of the administration is presented in Figure 6. All treated mice were euthanized in a CO_2_ chamber using a compressed gas cylinder with a pressure regulator and a flowmeter. Brain tissues were collected and stored at −75 °C for later analyses.

### 4.5. Infarct Volume Measurement

Pups were euthanized in a CO_2_ chamber at 7 days after UCB and EPO administration. Infarct volume was assessed by hematoxylin and eosin (H&E) (Sigma, St. Louis, MO, USA). The pups’ brains were fixed in 4% paraformaldehyde for 24 h, followed by 30% sucrose, until brains submerged completely (Each *n* = 3 per group). Using a freezing microtome, brains were cut into 25 μm thick sections. Three slices were chosen for subsequent H&E staining. The ipsilateral and contralateral hemispheres areas were measured using the ImageJ program (National Institute of Health, Bethesda, MD, USA). After correcting edema, the volumes of infarction were calculated as follows: Total volume of contralateral hemisphere (Total volume of the ipsilateral hemisphere–Average volume of 3 measurements of infarct).

### 4.6. Microarray Analysis

All treated mice were euthanized in a CO_2_ chamber. Brain tissues were collected and stored at −75 °C for later microarray analyses. We extracted total RNA from the ipsilateral hemisphere tissue of the mouse. Each treated brain sample was analyzed in comparison with a sham sample, HIE sample, UCB and EPO, and UCB + EPO sample, 7 days after inducing HIE. The microarray processing was performed using Affymetrix microarrays, with standard procedures (GeneChip^®^ Mouse Gene 2.0 ST Array, Affymetrix, Santa Clara, CA, USA). In brief, the sense cDNA was then fragmented and biotin-labeled with TdT (terminal deoxynucleotidyl transferase) using the GeneChip WT Terminal labeling kit. Approximately 5.5 μg of labeled DNA target was hybridized to the Affymetrix GeneChip^®^ Mouse Gene 2.0 ST Array at 45 °C for 16 h. Hybridized arrays were washed and stained on a GeneChip Fluidics Station 450 and scanned on a GCS3000 Scanner (Affymetrix, Santa Clara, CA, USA). Signal values were computed using Affymetrix^®^ GeneChip™ Command Console Software. Array data analysis and export processing were performed using Affymetrix^®^ GeneChip Command Console^®^ Software (AGCC) [58]. All microarray data generated in this study were deposited in the Gene Expression Omnibus database (GSE196570).

### 4.7. Principal Component Analysis

In the data dimension with multiple variables, the most important components were extracted in order. We then performed PCA, a linear dimensionality reduction technique in which fewer components containing most of the original information are represented by new variables [25]. R software (ver. 4.0.5) was used to determine the similarity of status or characteristics between samples.

### 4.8. Analysis of Differentially Expressed Genes and Hierarchical Clustering

We exported the result with gene-level RMA analysis, and the differentially expressed genes (DEGs) were analyzed using the R software package. The fold change (FC), calculated using |log2FC| ≥ 1, was considered for DEGs of interest; the *p*-value was not considered because there was only one sample in each group. Based on the gene list from the DEG results, analysis was performed using the Heatmap3 R package (ver. 1.1.9) to visualize the gene expression pattern of each sample. To evaluate the similarity of gene expression patterns between samples, distance and score calculations were performed with unsupervised clustering using the Euclidean method [59].

### 4.9. Gene Enrichment Analysis

Gene enrichment analysis with a BP database was performed using the ClueGO (ver. 2.5.6) package of Cytoscape (ver. 3.8.2) software, and using the web-based tool g:Profiler [22], the BP, MF, and KEGG were assessed to identify the gene ontology terms.

Pathway enrichment analysis for genes demonstrated the synergistic therapeutic effects of EPO, UCB, and a combination of EPO and UCB in hypoxic–ischemic encephalopathy-treated mouse tissue. The significance cutoff was *p*-value < 0.05, and the Kappa score (K = 0.4) was calculated to identify associations.

### 4.10. Total RNA Extraction and Quantitative Real-Time Polymerase Chain Reaction (qRT-PCR)

All treated mice were euthanized in a CO_2_ chamber. Brain tissues were collected and stored at −75 °C for later analyses. The cellular RNA from pup brains was extracted with TRIzol (ThermoFisher scientific, USA) (Each *n* = 8 per group). The cDNA was synthesized through reverse transcription (AccuPower PCR Premix, Bioneer, Chungbuk, Korea) and amplified by real-time PCR using a CFX Connect Real-Time PCR Detection System (Bio-Rad Laboratories, Hercules, CA, USA) and an SYBR Green detection system (Bioneer, Chungbuk, Korea). The primer sequences are presented in Table 2. mRNA expression levels were normalized to those of glyceraldehyde-3-phosphate dehydrogenase and were expressed relative to the average of all delta cycle threshold values in each sample using the cycle threshold method.

### 4.11. Statistical Analysis

All results are presented as the mean ± standard error of the mean; the overall significance of the experimental data was examined by one-way ANOVA and Tukey’s post hoc tests for multiple comparison analysis using Prism 8.0 (GraphPad Software Inc., San Diego, CA, USA). Differences between groups were considered statistically significant at *p* < 0.05.

## Figures and Tables

**Table 1:** The up-regulated genes of the UCB and EPO combination treatment were identified by Gene ontology (GO) enrichment analysis. The terms show the relevant pathways and genes after UCB and EPO combination treatment in HIE.

Biological Category	Term	−log10 (adjusted *p*-Value)	Associated Genes
Cellular structure	positive regulation of actin cytoskeleton reorganization	1.400660	Baiap2l1
Dopamine related pathways	response to amphetamine	1.504507	Nr4a2
	response to amine	1.923929	Nr4a2
	response to insecticide	1.524137	Nr4a2
	epithalamus development	1.711028	Nr4a2
	habenula development	1.711028	Nr4a2
	regulation of dopamine metabolic process	1.504507	Nr4a2
	regulation of catecholamine metabolic process	1.923929	Nr4a2
	response to corticotropin-releasing hormone	1.524137	Nr4a2
	regulation of respiratory gaseous exchange	1.641285	Nr4a2
	general adaptation syndrome	1.854837	Nr4a2
	cellular response to corticotropin-releasing hormone stimulus	1.524137	Nr4a2
Immune response	macrophage cytokine production	1.321089	Sema7a
	regulation of macrophage cytokine production	1.321089	Sema7a
	positive regulation of myeloid leukocyte cytokine production involved in immune response	1.504507	Sema7a
zinc protein pathways	zinc ion transmembrane transporter activity	1.641285	Slc30a3
	detoxification of zinc ion	1.711028	Slc30a3
	sequestering of zinc ion	1.482841	Slc30a3
	sequestering of metal ion	1.325929	Slc30a3
	regulation of sequestering of zinc ion	1.524137	Slc30a3
	zinc ion import into organelle	1.854837	Slc30a3
	zinc ion import into synaptic vesicle	1.854837	Slc30a3
	stress response to zinc ion	1.711028	Slc30a3

**Table 2:** Primers used in this study.

Gene	Forward Primer (5′-3′)	Reverse Primer (5′-3′)
Nurr1	GTGTTCAGGCGCAGTATGG	TGGCAGTAATTTCAGTGTTGGT
Nur77	TTGAGTTCGGCAAGCCTACC	GTGTACCCGTCCATGAAGGTG
NOR1	AGGATTCACTGATCTCCCCAA	GATGCAGGACAAGTCCATTGC
DAT	AAATGCTCCGTGGGACCAATG	GTCTCCCGCTCTTGAACCTC
VMAT	ATGCTGCTCACCGTCGTAG	GGACAGTCGTGTTGGTCACAG
Wnt1	GGTTTCTACTACGTTGCTACTGG	GGAATCCGTCAACAGGTTCGT
Wnt3	CTCGCTGGCTACCCAATTTG	CTTCACACCTTCTGCTACGCT
Wnt5	CAACTGGCAGGACTTTCTCAA	CATCTCCGATGCCGGAACT
β-catenin	ATGCAGCCGGACAGAAAAGC	CTTGCCACTCAGGGAAGGA
GSK-3β	TGGCAGCAAGGTAACCACAG	CGGTTCTTAAATCGCTTGTCCTG

## Data Availability

The data presented in this study are available upon request from the corresponding author.

## Supplementary Materials

The following supporting information can be downloaded at: https://www.mdpi.com/article/10.3390/ijms23052900/s1.

## References

[1] Lai M.-C., Yang S.-N. (2010). Perinatal hypoxic-ischemic encephalopathy. J. Biomed. Biotechnol..

[2] Allen K.A., Brandon D.H. (2011). Hypoxic ischemic encephalopathy: Pathophysiology and experimental treatments. Newborn Infant Nurs. Rev..

[3] Vannucci S.J., Hagberg H. (2004). Hypoxia–ischemia in the immature brain. J. Exp. Biol..

[4] Tetorou K., Sisa C., Iqbal A., Dhillon K., Hristova M. (2021). Current Therapies for Neonatal Hypoxic–Ischaemic and Infection-Sensitised Hypoxic–Ischaemic Brain Damage. Front. Synaptic Neurosci..

[5] Fatemi A., Wilson M.A., Johnston M.V. (2009). Hypoxic-ischemic encephalopathy in the term infant. Clin. Perinatol..

[6] Michael-Asalu A., Taylor G., Campbell H., Lelea L.-L., Kirby R.S. (2019). Cerebral palsy: Diagnosis, epidemiology, genetics, and clinical update. Adv. Pediatrics.

[7] Gonzales-Portillo G.S., Reyes S., Aguirre D., Pabon M.M., Borlongan C.V. (2014). Stem cell therapy for neonatal hypoxic-ischemic encephalopathy. Front. Neurol..

[8] Sato Y., Ueda K., Kondo T., Hattori T., Mikrogeorgiou A., Sugiyama Y., Suzuki T., Yamamoto M., Hirata H., Hirakawa A. (2018). Administration of bone marrow-derived mononuclear cells contributed to the reduction of hypoxic-ischemic brain injury in neonatal rats. Front. Neurol..

[9] Zheng Z., Zhang L., Qu Y., Xiao G., Li S., Bao S., Lu Q.R., Mu D. (2018). Mesenchymal stem cells protect against hypoxia-ischemia brain damage by enhancing autophagy through brain derived neurotrophic factor/mammalin target of rapamycin signaling pathway. Stem Cells.

[10] Eggenberger S., Boucard C., Schoeberlein A., Guzman R., Limacher A., Surbek D., Mueller M. (2019). Stem cell treatment and cerebral palsy: Systemic review and meta-analysis. World J. Stem Cells.

[11] Yang L., Qian J., Yang B., He Q., Wang J., Weng Q. (2021). Challenges and Improvements of Novel Therapies for Ischemic Stroke. Front. Pharmacol..

[12] Bang O.Y., Kim E.H., Cha J.M., Moon G.J. (2016). Adult stem cell therapy for stroke: Challenges and progress. J. Stroke.

[13] Shin Y.-K., Cho S.-R. (2016). Exploring erythropoietin and G-CSF combination therapy in chronic stroke patients. Int. J. Mol. Sci..

[14] Larpthaveesarp A., Pathipati P., Ostrin S., Rajah A., Ferriero D., Gonzalez F.F. (2021). Enhanced Mesenchymal Stromal Cells or Erythropoietin Provide Long-Term Functional Benefit After Neonatal Stroke. Stroke.

[15] Esneault E., Pacary E., Eddi D., Freret T., Tixier E., Toutain J., Touzani O., Schumann-Bard P., Petit E., Roussel S. (2008). Combined therapeutic strategy using erythropoietin and mesenchymal stem cells potentiates neurogenesis after transient focal cerebral ischemia in rats. J. Cereb. Blood Flow Metab..

[16] Kang M., Min K., Jang J., Kim S.C., Kang M.S., Jang S.J., Lee J.Y., Kim S.H., Kim M.K., An S.A. (2015). Involvement of immune responses in the efficacy of cord blood cell therapy for cerebral palsy. Stem Cells Dev..

[17] Hwang S., Choi J., Kim M. (2019). Combining human umbilical cord blood cells with erythropoietin enhances angiogenesis/neurogenesis and behavioral recovery after stroke. Front. Neurol..

[18] Cao Q.-Q., Li S., Lu Y., Wu D., Feng W., Shi Y., Zhang L.-P. (2021). Transcriptome analysis of molecular mechanisms underlying facial nerve injury repair in rats. Neural Regen. Res..

[19] Cai Y., Zhang Y., Ke X., Guo Y., Yao C., Tang N., Pang P., Xie G., Fang L., Zhang Z. (2019). Transcriptome sequencing unravels potential biomarkers at different stages of cerebral ischemic stroke. Front. Genet..

[20] Jiang X., Qin W., Wu J., Xiao J., Zhong Y., Yuan C., Yuan Q. (2021). Transcriptome analysis and differentially expressed gene screening for hypoxic-ischemic brain damage in rats treated with acupuncture. Acupuncture.

[21] Juul S.E., Beyer R.P., Bammler T.K., McPherson R.J., Wilkerson J., Farin F.M. (2009). Microarray analysis of high-dose recombinant erythropoietin treatment of unilateral brain injury in neonatal mouse hippocampus. Pediatric Res..

[22] Mary-Huard T., Daudin J.-J., Baccini M., Biggeri A., Bar-Hen A. (2007). Biases induced by pooling samples in microarray experiments. Bioinformatics.

[23] Schurch N.J., Schofield P., Gierliński M., Cole C., Sherstnev A., Singh V., Wrobel N., Gharbi K., Simpson G.G., Owen-Hughes T. (2016). How many biological replicates are needed in an RNA-seq experiment and which differential expression tool should you use?. RNA.

[24] Reimand J., Isserlin R., Voisin V., Kucera M., Tannus-Lopes C., Rostamianfar A., Wadi L., Meyer M., Wong J., Xu C. (2019). Pathway enrichment analysis and visualization of omics data using g: Profiler, GSEA, Cytoscape and EnrichmentMap. Nat. Protoc..

[25] Holland S.M. (2008). Principal Components Analysis (PCA).

[26] Cai H., Chen H., Yi T., Daimon C.M., Boyle J.P., Peers C., Maudsley S., Martin B. (2013). VennPlex–a novel Venn diagram program for comparing and visualizing datasets with differentially regulated datapoints. PLoS ONE.

[27] Rodriguez M.Z., Comin C.H., Casanova D., Bruno O.M., Amancio D.R., Costa L.d.F., Rodrigues F.A. (2019). Clustering algorithms: A comparative approach. PLoS ONE.

[28] Tilford C.A., Siemers N.O. (2009). Gene set enrichment analysis. Protein Networks and Pathway Analysis.

[29] Holm S. (1979). A simple sequentially rejective multiple test procedure. Scand. J. Stat..

[30] Perlmann T., Wallén-Mackenzie Å. (2004). Nurr1, an orphan nuclear receptor with essential functions in developing dopamine cells. Cell Tissue Res..

[31] Frasch M.G., Schulkin J., Metz G.A., Antonelli M. (2017). Animal models of fetal programming: Focus on chronic maternal stress during pregnancy and neurodevelopment. Animal Models for the Study of Human Disease.

[32] Luo S.X., Huang E.J. (2016). Dopaminergic neurons and brain reward pathways: From neurogenesis to circuit assembly. Am. J. Pathol..

[33] Fei Y.-x., Zhu J.-p., Zhao B., Yin Q.-y., Fang W.-r., Li Y.-m. (2020). XQ-1H regulates Wnt/GSK3β/β-catenin pathway and ameliorates the integrity of blood brain barrier in mice with acute ischemic stroke. Brain Res. Bull..

[34] Li P., Zhang Y., Liu H. (2019). The role of Wnt/β-catenin pathway in the protection process by dexmedetomidine against cerebral ischemia/reperfusion injury in rats. Life Sci..

[35] Armoskus C., Moreira D., Bollinger K., Jimenez O., Taniguchi S., Tsai H.-W. (2014). Identification of sexually dimorphic genes in the neonatal mouse cortex and hippocampus. Brain Res..

[36] Wang L., Shilatifard A. (2019). UTX mutations in human cancer. Cancer Cell.

[37] Choi J.I., Choi J.-W., Shim K.-H., Choung J.S., Kim H.-J., Sim H.R., Suh M.R., Jung J.E., Kim M. (2021). Synergistic Effect in Neurological Recovery via Anti-Apoptotic Akt Signaling in Umbilical Cord Blood and Erythropoietin Combination Therapy for Neonatal Hypoxic-Ischemic Brain Injury. Int. J. Mol. Sci..

[38] Singh M., Vaishnav P.K., Dinda A.K., Mohanty S. (2020). Evaluation of priming efficiency of forskolin in tissue-specific human mesenchymal stem cells into dopaminergic neurons: An in vitro comparative study. Cells.

[39] Yanagisawa D., Qi M., Kim D.-h., Kitamura Y., Inden M., Tsuchiya D., Takata K., Taniguchi T., Yoshimoto K., Shimohama S. (2006). Improvement of focal ischemia-induced rat dopaminergic dysfunction by striatal transplantation of mouse embryonic stem cells. Neurosci. Lett..

[40] Chen X.X., Qian Y., Wang X.P., Tang Z.W., Xu J.T., Lin H., Yang Z.Y., Song X.B., Lu D., Guo J.Z. (2018). Nurr1 promotes neurogenesis of dopaminergic neuron and represses inflammatory factors in the transwell coculture system of neural stem cells and microglia. CNS Neurosci. Ther..

[41] Gao H., Wang D., Jiang S., Mao J., Yang X. (2021). NF-κB is negatively associated with Nurr1 to reduce the inflammatory response in Parkinson’s disease. Mol. Med. Rep..

[42] Yu S., Doycheva D.M., Gamdzyk M., Yang Y., Lenahan C., Li G., Li D., Lian L., Tang J., Lu J. (2021). Activation of MC1R with BMS-470539 attenuates neuroinflammation via cAMP/PKA/Nurr1 pathway after neonatal hypoxic-ischemic brain injury in rats. J. Neuroinflammation.

[43] Li X., Li H., Bi J., Chen Y., Jain S., Zhao Y. (2012). Human cord blood-derived multipotent stem cells (CB-SCs) treated with all-trans-retinoic acid (ATRA) give rise to dopamine neurons. Biochem. Biophys. Res. Commun..

[44] Frazzini V., Rockabrand E., Mocchegiani E., Sensi S. (2006). Oxidative stress and brain aging: Is zinc the link?. Biogerontology.

[45] Xie X., Peng L., Zhu J., Zhou Y., Li L., Chen Y., Yu S., Zhao Y. (2017). miR-145-5p/Nurr1/TNF-α signaling-induced microglia activation regulates neuron injury of acute cerebral ischemic/reperfusion in rats. Front. Mol. Neurosci..

[46] Jankovic J., Chen S., Le W. (2005). The role of Nurr1 in the development of dopaminergic neurons and Parkinson’s disease. Prog. Neurobiol..

[47] Tito A.J., Zhang S. (2016). Characterization of Vesicular Monoamine Transporter 2 and its Role in Parkinson’s Disease Pathogenesis Using Drosophila. Ph.D. Thesis.

[48] Jia L., Piña-Crespo J., Li Y. (2019). Restoring Wnt/β-catenin signaling is a promising therapeutic strategy for Alzheimer’s disease. Mol. Brain.

[49] Fairbanks S.L., Young J.M., Nelson J.W., Davis C., Koerner I.P., Alkayed N.J. (2012). Mechanism of the sex difference in neuronal ischemic cell death. Neuroscience.

[50] Hu C., Bai X., Liu C., Hu Z. (2019). Long noncoding RNA XIST participates hypoxia-induced angiogenesis in human brain microvascular endothelial cells through regulating miR-485/SOX7 axis. Am. J. Transl. Res..

[51] Oorschot D.E., Sizemore R.J., Amer A.R. (2020). Treatment of neonatal hypoxic-ischemic encephalopathy with erythropoietin alone, and erythropoietin combined with hypothermia: History, current status, and future research. Int. J. Mol. Sci..

[52] Herz J., Köster C., Reinboth B.S., Dzietko M., Hansen W., Sabir H., van Velthoven C., Bendix I., Felderhoff-Müser U. (2018). Interaction between hypothermia and delayed mesenchymal stem cell therapy in neonatal hypoxic-ischemic brain injury. Brain Behav. Immun..

[53] Park W.S., Sung S.I., Ahn S.Y., Yoo H.S., Sung D.K., Im G.H., Choi S.J., Chang Y.S. (2015). Hypothermia augments neuroprotective activity of mesenchymal stem cells for neonatal hypoxic-ischemic encephalopathy. PLoS ONE.

[54] Zhou K.Q., Davidson J.O., Bennet L., Gunn A.J. (2020). Combination treatments with therapeutic hypothermia for hypoxic-ischemic neuroprotection. Dev. Med. Child Neurol..

[55] Min K., Song J., Lee J.H., Kang M.S., Jang S.J., Kim S.H., Kim M. (2013). Allogenic umbilical cord blood therapy combined with erythropoietin for patients with severe traumatic brain injury: Three case reports. Restor. Neurol. Neurosci..

[56] Novak I., Paton M.C., Finch-Edmondson M. (2021). Research update on the state of the evidence for stem cell and regenerative medicine in cerebral palsy. Arch. Stem Cell Ther..

[57] Lan K.-M., Tien L.-T., Cai Z., Lin S., Pang Y., Tanaka S., Rhodes P.G., Bhatt A.J., Savich R.D., Fan L.-W. (2016). Erythropoietin ameliorates neonatal hypoxia-ischemia-induced neurobehavioral deficits, neuroinflammation, and hippocampal injury in the juvenile rat. Int. J. Mol. Sci..

[58] Irizarry R.A., Hobbs B., Collin F., Beazer-Barclay Y.D., Antonellis K.J., Scherf U., Speed T.P. (2003). Exploration, normalization, and summaries of high density oligonucleotide array probe level data. Biostatistics.

[59] Zhao S., Guo Y., Sheng Q., Shyr Y. (2014). Advanced heat map and clustering analysis using heatmap3. BioMed Res. Int..

